# The Southern Surgical and Gynecological Association

**Published:** 1893-12

**Authors:** 


BUFFALO MEDICAL AND SURGICAL JOURNAL
A MONTHLY REVIEW OF MEDICINE AND SURGERY.

*EDITORS:*
THOMAS LOTHROP, M. D. - - WM. WARREN POTTER, M. D.

All communications, whether of a literary or business character, should be addressed to the managing editor: 284 FRANKLIN STREET, BUFFALO, N. Y.

VOL. XXXIII. DECEMBER, 1893. No. 5.

### THE SOUTHERN SURGICAL AND GYNECOLOGICAL ASSOCIATION.

THE sixth annual meeting of this association was held in New Orleans, on November 14, 15, and 16, 1893, and it may be justly characterized as one of the best medical meetings of the year. This association is noted for its good work, but it seems to have fairly outdone itself during its late annual gathering. Dr. Bedford Brown, the President, is a representative Southern surgeon, and an experienced man in the conduct of medical societies. It was fortunate for the association that Dr. Brown's term of service occurred during the year that the meeting was held in New Orleans. In his response to the address of welcome, among other things, he said :

"I am sincerely glad that I came to New Orleans. I am glad to know the gentlemen of the profession of this city. I have long heard of the high character, the great skill, and high order of intelligence of the medical men here, and I congratulate the association that we have met in this grand, old, historic city, and that we are permitted to associate with them on common grounds and on terms of friendship and unity. Gentlemen, I thank you."

His speech was greeted with prolonged applause, and the association then fairly got down to its work. The first paper was an address on the Life and Character of Ephraim McDowell, of Kentucky, by Dr. L. S. McMurtry, of Louisville. Dr. McMurtry is singularly adapted to the task which he assumed by vote of the association on this occasion, for he himself is a native of the city of Danville, where McDowell first introduced to the world abdominal surgery by the performance of an ovariotomy. Dr. McMurtry is thoroughly familiar with the subject on which he discoursed, having been secretary of the McDowell Association, and one of the prime movers in erecting the monument to the memory of the great surgeon, which stands in the center of the plot set apart therefor in the city of Danville. We shall anxiously await a full report of Dr. McMurtry's address.

The association was the guest of the Boston Club and the Pickwick Club during its stay in New Orleans, and a special entertainment was given by the French Opera Company for their enjoyment, the opera of "Faust" having been selected for the evening. On Wednesday evening, November 15th, the association was entertained at a reception at the St. Charles Hotel, given by the medical profession of New Orleans.

Dr. Joseph Price was especially invited to operate in the amphitheatre of the Charity Hospital on Wednesday morning, at 8.30 o'clock, when a large audience assembled to witness the technique of this expert and successful abdominal surgeon. The attendance upon this annual meeting was uncommonly large, the scientific work done was enormous, the debates were spirited and instructive, and the work of the stenographer, Dr. Whitford, was especially heavy. The next volume of transactions will be an interesting one, and deserves to be in the hands of every Southern physician, as well as those who may be unfortunate enough to reside on the north side of Mason and Dixon's line.

Dr. Brown was especially felicitous in his annual address, which secured the attention and the approbation of his auditors as testified by generous plaudits. He gave the history of the organization of the association, and traced its eventful career down to the present time. He said, *inter alia*, that the origin of the Southern Surgical and Gynecological Association was based on the necessity of the times. Its object was the promotion of the practice of scientific surgery and gynecology in the South, and its aim was the development of the surgical talent and learning of the Southern profession. Forming, he said, an opinion on his own individual experience and by that of others, he was persuaded that no member who had followed the progress of the association closely, and who had taken assiduous part in the proceedings, had failed in having his knowledge of the science of surgery and gynecology expanded, or his ideas enlarged by the able papers and discussions that have characterized the transactions. He defended the association from the charge of sectionalism in the following words:

In conclusion, I desire to make some passing allusion to the alleged sectional character of our title and purpose, "The Southern Surgical and Gynecological Association." Those who founded this association never entertained the remotest idea or intention of organizing it, or using it, for sectional purposes. They believed that the necessities of the medical profession of the South demanded and required such an institution, based upon broad, liberal, and democratic principles, open to merit, talent, and character for the encouragement of medical progress in that section.

No, we are of all people the least sectional. We are a band of workers linked together, working with one mind, with the single purpose, the noble object alone of promoting the sciences of surgery and gynecology. We know no politics, no political distinctions. We do not even know the political opinions entertained by each other. We are composed of ardent workers in search of scientific knowledge from the far North, the distant West, the East, and the South, that mingle together on perfect terms of equality, in social pleasure, and in a spirit of kindliness and mutual confidence. There are those here mingling together in friendly intercourse who took part in the great struggle and who stood arrayed against each other in bloody array, now united in friendship and engaged in the humane office of devising means for alleviating human suffering and prolonging human life. The scene, as it presents itself to my mind, of men with one single and grand and glorious object, working together harmoniously, congenially, trustingly, forgetting all differences of opinion, political and social, each ready to make sacrifices for the other and for the good of the cause, presents a sight truly noble and worthy of the greatest admiration. To those illustrious and distinguished men who have, at our invitation, come from the great cities of the north—Boston, New York, Philadelphia, Buffalo, Chicago, Cincinnati, and St. Louis—and united their destinies with ours, and are sharing with us our toils and responsibilities, I have only words of admiration. commendation, and kindliness.

The concluding sentences of his splendid address were words of caution towards faithfully guarding the honor of the association, maintaining its high standard of purity, and its great strength of character. When the erudite and venerable President had concluded his address, the plaudits that rang in his ears must have nearly deafened him. After the usual complimentary vote of thanks, a resolution was offered by Dr. Kollock, of South Carolina, instructing that 500 copies of the President's address be struck off for distribution among the members. We believe the careful perusal of this valuable paper will be of great service to this and similar associations.

A long list of new Fellows were read, and it was unanimously voted to hold the next meeting at Charleston, S. C. The following-named officers were elected for the ensuing year: President, Cornelius Kollock, M. D., of Cheraw, S. C.; Vice-president, A. B. Miles, M. D., New Orleans; Secretary, W. E. B. Davis, M. D., Birmingham, Ala.; Treasurer, J. B. S. Holmes, Rome, Ga.; to fill vacancy in Council, Bedford Brown, M. D., of Alexandria, Va.

Dr. L. S. McMurtry, of Louisville, then took the floor, and presented resolutions expressing the thanks of the association for the courtesies and hospitalities so gracefully and generously extended by the Committee of Arrangements, and from the members of the profession in New Orleans; to the ladies who graced with their presence the reception on Wednesday night; to the daily press for its very complete report; to the Boston Club and the Pickwick Club for courtesies and hospitalities enjoyed. Dr. McMurtry supported his resolutions with the following remarks:

Mr. President—The scientific work provided for the sixth annual meeting of the Southern Surgical and Gynecological Association has been completed. Within a few hours the trains will be carrying our Fellows to their distant homes and their respective fields of labor. The valuable papers, discussions, and demonstrations, which have made this meeting a conspicuous one in its history, will be presented to the profession in the annual volume of transactions. Before the gavel falls we would express our appreciation of the hospitality and courtesies we have received from our confrères in this Crescent City of the South, a hospitality so cordial and generous, a courtesy so spontaneous and graceful, as never to be surpassed or forgotten. The bright sunshine of this genial clime, where the air bears abroad the fragrance of the magnolia and orange blossom, has seemed only a natural and harmonious element in these three days of pleasing labor and delightful recreation. It is all beautiful, and will remain a happy memory.

It is most appropriate that this organization, to advance and diffuse surgical science in the South, should convene in this Southern metropolis, where was founded, in the century's early decades, one of America's most famous schools of medicine. To conduct our deliberations in this beautiful Temple of Medicine, fresh from the hands of the builder, and so recently dedicated to science, has been an inspiration. Here, within a square of us, was the home of Warren Stone and the site of his private hospital, the first ever founded in America by individual enterprise. The surgical career of Stone is one of the glories of this university. A great teacher, an original and courageous operator, he made a lasting impression on Southern surgery. If the elder Gross has the place in our surgical annals occupied in the history of European surgery by Sir Astley Cooper, then was Stone our Velpeau. To Stone, more than any other, is due the gratitude of the profession for the splendid educational privileges we have witnessed in the Charity Hospital. There, in those wards, taught for years the scholarly and sagacious clinician, whom many now present knew and loved, Samuel M. Bemiss. Who that ever heard the gifted Hawthorn, cut down by the reaper in his prime, can forget the clinical lessons ? If in Stone we found here the prototype of the blacksmith surgeon of the French school of surgery, surely in Hawthorn we had the American Trosseau. These halls have so forcibly and constantly reminded us of the life and labors of Richardson and Logan, so recently gone over to the silent majority, that we can scarcely realize that these two eminent Southern surgeons are no longer here. These eminent teachers are at rest, the lot universally meted out to mankind, but their great work goes on. We know that the standards they bore are safe in the hands of our confrères, and will be borne ever onward in the front ranks of advancing science.

These thoughts, Mr. President, have been suggested by the occasion, and it is our wish that our confrères be assured of our keen appreciation of their courtesy. We hope that our visit here may strengthen the professional ties among our members, and cement those ties by the stronger bonds of personal friendship and mutual esteem.

The speech was received with prolonged applause, and the resolution adopted most heartily.

President Brown took the floor, and, on his own behalf, said a few appropriate words on the hospitality with which he and his fellows had been received in New Orleans.

We have devoted considerable space to this meeting, because we regard it as a timely theme to discourse upon. There are so many medical associations that are useless, or without much value, that we feel it a pleasure to commend the work of a body like the Southern Surgical and Gynecological Association, and to extend its fame and methods as far as lies in our power so to do.

